# Supplementary material for: Too much to process? Exploring the relationships between communication and information overload and videoconference fatigue
Source: PLoS One. 2024 Dec 4;19(12):e0312376. doi: 10.1371/journal.pone.0312376 (PMC11616880; doi:10.1371/journal.pone.0312376)
Supplement: Attachment — Submitted filename: Response to Reviewers Sep 24.docx [file pone.0312376.s002.docx]

Journal Requirements:
Please review your reference list to ensure that it is complete and correct. If you have cited papers that have been retracted, please include the rationale for doing so in the manuscript text, or remove these references and replace them with relevant current references. Any changes to the reference list should be mentioned in the rebuttal letter that accompanies your revised manuscript. If you need to cite a retracted article, indicate the article’s retracted status in the References list and also include a citation and full reference for the retraction notice.
_______________________________

Reviewers' comments:

Reviewer's Responses to Questions

**Comments to the Author**
Reviewer #1: Dear Authors,

Thank you for the opportunity to review your manuscript titled "Too much to process? Exploring the relationships between communication and information overload and videoconference fatigue." Your study addresses a highly relevant and timely issue in the context of modern communication, particularly as the world increasingly relies on videoconferencing for both educational and professional interactions. Overall, the manuscript is well-structured, methodologically sound, and written in clear and standard English.

**Response:** Thank you for your positive comments.

Reviewer #2: Dear authors,
Thank you for considering my previous suggestions and for addressing all comments I made in the original manuscript. All comments have been addressed accordingly and are acceptable.
I, however, would strongly recommend that the revised manuscript to go through another round of proofreading to enhance the clarity of the sentences, especially for the newly added sentences.

Please kindly refer to some examples below:
- Line 266 (page 11): 'who under 21 old'
- Line 268 (page 12): 'all participants had the right to withdraw' --> to consider rephrasing the sentence to 'all participants were made aware about their rights to withdraw'
- Line 309 (page 15): 'As Singapore is a multiracial society' --> to consider using the word 'country' instead of 'society'.

**Response:** Thank you very much for your suggestions. We have proofread the article closely and made edits to improve the clarity of the manuscript. In addition, we have made the following edits based on the examples you raised:

“Participation was entirely voluntary. All respondents provided their informed consent prior to data collection, and consent was also obtained from the parents of respondents who were under the age of 21.”

“During the experiment, all participants were made aware about their rights to withdraw at any time or choose not to answer any questions that made them feel uncomfortable.

“As Singapore is a multiracial country”
